# Benefits of Multidimensional Measures of Child Well Being in China

**DOI:** 10.3390/ijerph14111349

**Published:** 2017-11-06

**Authors:** Shirley Gatenio Gabel, Yiwei Zhang

**Affiliations:** Graduate School of Social Service, Fordham University, New York, NY 10458, USA; yzhang347@fordham.edu

**Keywords:** child well-being, multidimensional well-being, multidimensional poverty, China, children, child indicators

## Abstract

In recent decades, measures of child well-being have evolved from single dimension to multidimensional measures. Multi-dimensional measures deepen and broaden our understanding of child well-being and inform us of areas of neglect. Child well-being in China today is measured through proxy measures of household need. This paper discusses the evolution of child well-being measures more generally, explores the benefits of positive indicators and multiple dimensions in formulating policy, and then reviews efforts to date by the Chinese government, researchers, and non-governmental and intergovernmental organizations to develop comprehensive multidimensional measures of child well-being in China. The domains and their potential interactions, as well as data sources and availability, are presented. The authors believe that child well-being in China would benefit from the development of a multidimensional index and that there is sufficient data to develop such an index.

## 1. Introduction

Until recently, measures of child well-being in society were sparse. Little was known beyond the income adequacy of children’s families in most countries until the 1990s. Assessments of how well a society cared for its children were largely measures of how poorly children were faring, i.e., we counted the number of children in poverty. For children, growing up in poverty can be damaging to children’s physical, emotional, and spiritual development, and yet, child poverty is too often not differentiated from poverty in general. Child poverty differs from adult poverty in that it has different causes and effects, and the impact of poverty during childhood can cause lifelong cognitive and physical impairments that are sometimes irreversible. This perpetuates the cycle of poverty across generations. Investing in all children is therefore critical for achieving our growth potential as humans. As our understanding of the causes contributing to well-being have deepened and widened, so has our ability to measure the factors contributing to children’s quality of life. In China today, child poverty is commonly defined as children living in income-poor households [1,2]. We consider the benefits of introducing multidimensional measures of child well-being in China as the country develops more sophisticated measures of poverty prevention and well-being by reviewing efforts to collect date and ending with recommendations to move forward. Rather than offering a specific index to measure child well-being in China, we share the knowledge gained about the types of indicators used in indices in other countries and the availability of data for indicators that could be used in a multi-dimensional index in China. We shy away from offering a specific index because measures of well-being should be contextualized by culture and informed by those who are being measured [3].

## 2. Evolution of Multi-Dimensional Measures of Child Well Being

In this section, we review the evolution of measuring child well-being globally, from single dimensional income measures of poverty or maltreatment to positivist approaches that measure multiple dimensions of children’s lives. Unless otherwise indicated, children are defined as those below the age of 18 years.

The momentum for developing indicators to monitor the quality of children’s lives only came to fruition in the late 1970s and grew from a broader social indicator movement of the decade [4]. Until then, the focus was on developing child indicators that captured child survival or what we consider today to be negative indicators, that is, indicators that measure how poorly children develop or how the lives of children need to be rectified (e.g., maltreatment, malnutrition, deaths). UNICEF, the World Bank, and domestic organizations such as Child Trends in the United States first published reports documenting the quality of life for children in 1979, followed by OECD reports and other individual country reports.

Over time, interest developed in measuring positive as well as negative indicators of children’s lives in all countries due to: the reconceptualization of childhood as a unique developmental period and more than a preparatory period for adulthood; changing paradigms incorporating multi-dimensions of healthy child development, and the advent of children’s rights igniting a shift away from an exclusive adult perspective on child well-being [5]. Rather than defining children’s well-being as the absence of negative or undesirable behaviors, positive indicators monitor children’s progress and supports in achieving the significant milestones of human development and societal achievements [6,7].

The view of childhood as a distinct period in development perhaps began in 1762 with Jean-Jacques Rousseau’s notion presented in *Emile* that children were inherently good rather than evil and who, if treated well, would mature into responsible adults. Gradually, modern society saw childhood as a distinct and critical period in human development. Initially, our nearly exclusive focus was on improving childhood experiences so that children would become successful adults. Over time, we moved from focusing on childhood as a preparatory period for adulthood (well-becoming) to focusing on the well-being of children for its own sake [4].

This shift was informed by the growth in theories reflecting multidimensional aspects of child development. These theories deepened our understanding of optimal development of children and adolescents using strengths-based approaches focused on facilitating the growth of children’s assets, behaviors, relationships and morals [8,9]. For example, the importance of environmental interactions was captured early on in Bronfenbrenner’s ecological model of human development that conceptualizes child development as engagement with four concentric circles of environmental influence over time and then furthered by the work of Brooks-Gunn and Vygotsky [10,11,12].

Paralleling the strength-based theories supporting children’s holistic development, is the idea that children as human beings have human rights. This has re-framed our notion of childhood as not only a period of well-becoming but also as childhood having value in of itself and children having the right to express their own needs, opinions and goals [13]. The 1989 UN Convention on the Rights of the Child (CRC) embodies these values and is the most widely ratified human rights treaty (ratified by 196 countries by 2017) (http://indicators.ohchr.org/). The CRC makes clear that society must act in the best interests of children, all children are to be protected and provided with the supports needed to maximize their development, and that children have the right to participate in decisions affecting their well-being. The Convention declares that children have the rights and freedoms inherent to all human beings, including adequate nutrition, health care, education, and freedom from abuse, violence, and exploitation. By asserting the right of children to express their own opinions, it is a society’s responsibility to accept children’s subjective perspectives on their own well-being and for children to act as reporters assessing their well-being [14]. Several countries have used the CRC to develop new frameworks of indicators to monitor child well-being at both national (e.g., Spain, Ireland, Italy, New Zealand) and international levels [15,16,17].

The recognition of multiple factors contributing to child well-being highlighted the inadequacy of measuring child well-being through an income-consumption lens only. Single dimension standards of poverty assume that an individual’s needs can be summed up by the individual’s level of income and minimizes health, educational, cultural, infrastructure, and other factors they may affect consumption needs.

Criticism of using income as a proxy for human welfare in the research literature goes back to at least the 1960s but it was Amartya Sen’s capability approach, expanded upon by Martha Nussbaum [18], that provided the foundation to the multidimensional approach [19]. Both Sen and Nussbaum proposed a multidimensional approach to poverty and well-being that provides an overarching picture of a society by moving beyond merely combining results from economic and social sectors. According to the capabilities approach, the well-being of individuals is the freedom of individuals to act upon developing their capabilities in interconnected domains. If one of the five freedoms (political, economic, social opportunities, transparency, and security) is compromised, the others will be affected as well. For example, a child living in conditions where sanitation is compromised is likely to have poor health and may be unable to learn at school. From a policy perspective, this entails developing public policy responses responding to the interconnections among the different dimensions of deprivation as well as the specific areas of deprivation.

This approach supported exploration away from uni-dimensional, negative measures of poverty toward positive, integrative indicators of multidimensional well-being among researchers and policymakers. Multidimensional poverty measures not only provide aggregate assessments of who is poor but can also be broken down to identify subgroups for whom deprivations are more severe or have achieved progress. By specifying the deprivation(s), it more accurately identifies the resources needed to overcome the deprivation.

Most recently, the worldwide adoption of the United Nation’s Sustainable Development Goals (SDGs) has reinforced interest in multidimensional measures of well-being for populations in general and for children specifically. The SDGs, also known as the 2030 Agenda for Sustainable Development, was officially adopted by world leaders in September 2015 and universally applies to all countries who must work toward ending all forms of poverty, fighting inequalities and tackling climate change, while ensuring that no one is left behind. While the SDGs are not legally binding, governments have the responsibility of establishing national implementation and monitoring frameworks for the achievement of the 17 goals by 2030. The SDGs recognize that goals such as ending poverty must go hand-in-hand with strategies to foster economic growth, employment and education and health care for all while tackling climate change and environmental protection.

Countries have the primary responsibility for follow-up and review of the progress in achieving the SDGs and quality, accessible and timely data collection is needed. Multidimensional measures of poverty/well-being are increasingly popular means of measuring this progress (http://indicators.report/targets/).

**Figure 1:**
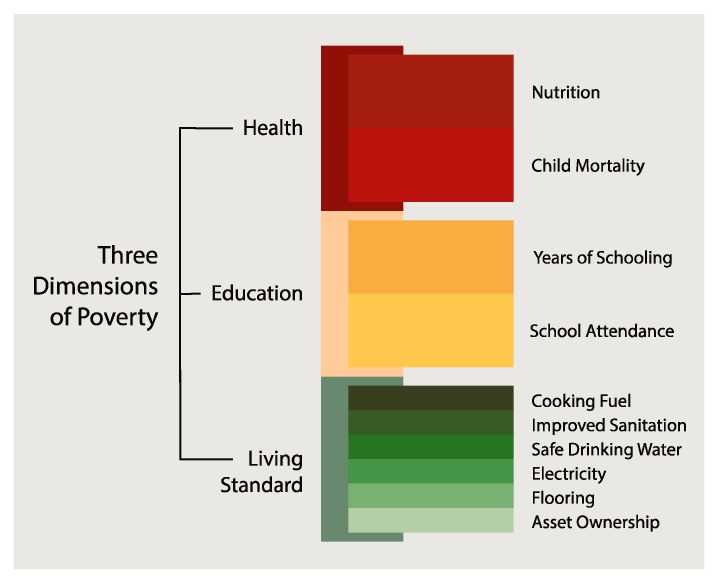
Global Multidimensional Poverty Index. (Source: http://www.ophi.org.uk/multidimensional-poverty-index/) [20].

As of 2017, 103 countries were using the Global Multidimensional Poverty Index (MPI) (Figure 1) to assess progress on SDGs. Oxford Poverty and Human Development Initiative (OPHI) together with the Human Development Report Office of UNDP developed the global MPI in 2010. OPHI updates the global MPI figures twice a year, and provides full detail of deprivations by each indicator, by subnational regions and groups, and over time. Based on the Alkire-Foster methodology, MPI measures household experiences of deprivation in any of ten areas of three main dimensions and a household is identified as poor if they suffer deprivations across one-third or more of the weighted indicators. The MPI is created by multiplying the percentage of the population who are poor by the intensity or average percentage of the weighted indicators. This helps identify acute poverty.

In 2013, 1.45 billion people from 103 countries were poor on multiple dimensions—nearly 27% of the population in these countries [20]. Interestingly, 72% of those considered poor using MPI live in middle income countries, and half of them are children aged 17 years or younger, most of whom live in South Asia and Sub-Saharan Africa. About 689 million, or 37%, of children are multidimensionally poor. Children are more likely to be poor than adults and are deprived in more indicators at the same time [20].

The 17 goals SDGs, its 169 targets, and its multidimensional measures of well-being are based on a fundamental assumption that well-being is a result of diverse factors coming together and deficits in one or more of these multifaceted approaches have the potential to stymie growth in other areas. Social policies must address how the existing social infrastructure has differential effects on different segments of the population. For example, collecting more taxes to pay for both stricter environmental regulation policies and extending health insurance may improve health outcomes but reduce household income. A multidimensional approach to policy development call for the net well-being effects to be estimated as the benefits may vary depending on the relative elasticities of income and health to these policy changes [21].

## 3. Poverty and Child Well-Being in China

Despite the shortcomings of uni-dimensional approaches, most countries employ a uni-dimensional, income-, or consumption-based measure to assess the well-being of its citizenry. In China, the official measure of poverty is an absolute indicator based on the cost of a minimum subsistence package consisting of food and non-food essential items [22]. Those with per capita net income or per capita consumption expenditure below the official poverty line are considered poor. China does not maintain (or share) poverty data for individual cities or even a national figure for urban poverty.

Poor children are typically defined as those living in a household with an income that is less than a designated income poverty line. Several studies have measured the child poverty rate using a uni-dimensional income indicator [1,23].

China also uses another proxy to estimate the number of poor children in China. Children who reside in households receiving assistance, dibao, are defined as poor. Dibao is the Minimum Living Standard Guarantee Scheme for low-income families primarily living in urban areas launched in the 1990s but recently was extended to rural areas as well in 2007 [24,25]. It tops off incomes to a minimum level set by local governments and benefits are typically low (e.g., the equivalent of $5 USD/day in Beijing).

Using dibao status to identify poor children is problematic because it omits those who are poor but may be ineligible for assistance for various reasons. For example, eligibility for dibao is determined based on the hukuo system. Hukuo is the household registration system in China. An urban dweller is only eligible for assistance if they continue to live in the urban area where they were registered to live. Migrant workers and their children coming from rural areas are excluded from the dibao scheme.

According to a World Bank report [26], the dibao program lowered the poverty gap by only 6.5 percent. Only 21 percent of poor households received dibao in 2010, and more than half of dibao recipients were above the poverty line [27]. Corruption plagues the program despite the great lengths local governments take to investigate the assets of dibao households [27].

Neither income-based or benefit recipient methods capture the full experiences and effects of children living below these income poverty lines or whether their basic rights have been fulfilled or not [28,29], and both of these approaches are based on negative indicators.

Less attention has been paid to capturing children’s well-being in China employing positive and multidimensional indicators such as children’s access to infrastructure and services that are critical for their development and future well-being.

**Figure 2:**
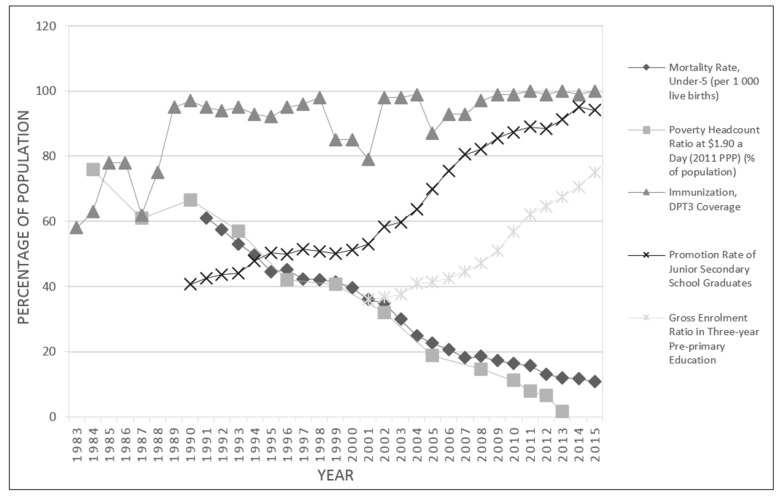
Child well-being trends in China. Data on mortality rate, under-5 are from National Bureau of Statistics of China [33]; Data on poverty headcount ratio at $1.90 a day are from The World Bank [30], available years include 1984, 1987, 1990, 1993, 1996, 1999, 2002, 2005, 2008, 2010–2013; Data on immunization, DPT3 coverage are from World Health Organization [32]; Data on promotion rate of junior secondary school graduates are from Department of Development and Planning, Ministry of Education (DDP) [34,35]; Data on gross enrolment ratio in three-year pre-primary education are from DDP [36].

If we look at indicators available, we see that the quality of life of Chinese children have improved dramatically since the 1980s in China (Figure 2). Income poverty ($1.90 a day in 2011) has decreased from 75.76% in 1984 to 1.85% in 2013 [30]. The total population in poverty has been reduced from 689 million in 1990 to 250 million in 2011 [31]. In 2015, the DPT3 immunization coverage reached around 99% [32], promotion rates of senior secondary rates are nearing universality from just over 40 percent in the late 1980s, and the under-5 mortality rate has declined to 10.7 children per 1000 live births [33].

The overall trends may mask the unequal gains among children. For example, in 2015, the under-5 mortality rate was 5.8‰ in urban China and 12.9‰ in rural areas [33]. Another significant disparity between rural and urban areas is the injury-related death rate. In 2014, the injury-related death was 12.74 per 100,000 children aged 1–17 years in urban areas compared to 17.03 for children in rural China [37]. For newborns, the disparity is starker: the rate of injury-related death was 84.53 per 100,000 children in rural areas vs. 20.64 for urban children [37].

Disparities also exist between different regions of China. For example, in rural China in 2013, the western region of China led with the highest rate for low birth weight (4.4%), followed by the eastern region (3.2%), and then the central region (2.1%) [38]. The pattern was slightly different in the urban setting, where low birth rates were 3.8% in central China and 2.8% in eastern China [38].

The number of children affected by migration is increasing rapidly and the rural-urban disparities and exclusionary policies and practices against migrant workers profoundly impact access to and the quality of education for children either left-behind or those who do migrate with their parents [39,40,41]. In 2010, approximately 106 million children were affected by migration, comprising about 38% of the total child population in China, which was almost 2.5 times of the number in 2000 [42,43,44]. About 24% of children attending nine-year compulsory education were affected by migration (both migrant with parents and left behind) [33]. However, data from the National Migrant Population Surveillance Survey shows that the proportion of children not attending compulsory education remains 4–5% each year from 2010–2015 for children migrant with parents [45]. Children affected by migration show less favorable emotional health, psychosocial, and educational well-being outcomes [46,47]. Over five percent of children migrant with parents and 6.31% of left-behind children, under the age of seven, do not complete the vaccines required by the national immunization plan [45], compared to around 99% immunization coverage rate for the overall child population in 2015 [32].

The hukuo system has left millions of migrant children living in income-precarious households with inadequate education, health care access, and poor housing and nutrition because they are unlikely to be eligible to receive social benefits from their parents’ employers. A multidimensional approach can help highlight rural and urban differences and other less recognized population vulnerabilities that uni-dimensional approaches typically do not reveal.

Using a multidimensional approach to measure child poverty in China would reveal differences within and between groups and regions by allowing the disaggregation of data by geographic area, ethnicity, gender or other social groups. Once group differences are noted, data can be broken down to show which deprivations are driving poverty within groups and the contribution of each indicator to overall poverty levels. This provides a clear map for coordinating the design and implementation of public programs and policies and lends itself to monitoring changes in poverty, severity and the composition of poverty over time using time series or panel data.

## 4. Multi-Dimensional Measures of Child Well-Being in China

A literature review on child well-being was conducted to learn how child well-being has been measured in major comparative studies. From this we learned the major dimensions commonly used and the corresponding indicators to measure multidimensional child well-being. We then conducted a literature review of the multidimensional indices on child well-being that explicitly focus on China or that include China among the countries in comparative studies. Table 1 summarizes five major studies that measure child well-being in China from a multidimensional perspective and compares them based on the eight dimensions we identify from the literature review and other comparative characteristics suggested by Fernandes, Mendes, and Teixeira [48]. The eight major dimensions identified to measure child well-being in China are: economic well-being; home environment: community and family contexts; safety/risk behaviors; health; education; emotional/subjective well-being; social relationships; and gender identity.

Two of the five studies, the Lau and Bradshaw [49] and Save the Children [50], are international comparisons. Lau and Bradshaw [49] compare child well-being in 13 Pacific Rim countries and regions, including China. Six dimensions and 21 components are identified and 46 indicators are measured. However, components of the education, deprivation and peer relationship data in China were not available. Another international effort is the Child Prosperity Index developed by Save the Children [50]. It measures the quality of children’s lives across the G20 countries, including China.

The other three studies focus on China in particular. In Chen, Yang, and Ren’s [51] Report on the State of Children in China, each indicator of child well-being is disaggregated by rural, urban, left behind, migrant, single/no parent family status. These are five living arrangements of children. Qi and Wu adopt the Alkire-Foster methodology using measures of deprivation to assess changes in multidimensional child poverty in China between 1989 and 2009 [29]. UNICEF published two reports since 2010 to measure child well-being in multiple areas, Children in China: An Atlas of Social Indicators [44].

Among the five studies, three of them [44,49,50] use aggregate data from national administrative data, international sampled surveys or databases (e.g., PISA, MISC, UNICEF, Speaking Out Survey, World Bank World Development Indicators, SWOC, WHO Mortality Database), and regional sampled surveys. Chen et al. [51] use a nationally representative bi-annual longitudinal dataset, China Family Panel Studies (CFPS). Qi and Wu [29] use the China Health and Nutrition Survey (CHNS), a time series dataset collected data in nine provinces since 1989. The age range differs across the five studies. Two studies [49,50] use indicators for children age 0–19. The rest three studies define children at age 0–17 [44], 0–15 [51], or 0–18 [29].

The Child Prosperity Index [50] is unique in that it includes a gender equality dimension—the gender inequality index developed by the UNDP Human Development Report is adopted—and uses two indicators—air pollution and carbon dioxide emissions—to measure a country’s environmental conditions. Unlike the other four studies, Qi and Wu [29] adopted deprivation indicators, which are all negative, in their multidimensional framework yet includes.

The children’s perspective is included in all but the Save the Children report. Lau and Bradshaw [49] highlight children’s views on their own well-being using data from a child-reported UNICEF survey (UNICEF, Speaking Out Survey 2001). Chen et al. [51] use data from the CFPS which includes a section on children’s view of their emotional health and social relationships that in particular focuses the well-being of children affected by migration. The UNICEF report [44] also highlights the conditions of children affected by migration. Chen et al. [51], analyze the data by the living arrangements of children (i.e., rural, urban, left behind, migrant, single/no parent family) and specifically compares rural-urban disparities in child well-being.

Findings from the two comparative studies show that China ranked mid-range on most indicators. Among the G20 countries, China ranks 11 and performs better than average across all dimensions compared to other middle-income countries [50]. China ranks sixth in the overall child well-being among the 13 Pacific Rim countries and regions and performs well in the subjective well-being and risk and safety dimensions [49]. The three studies whose sole focus is on China, indicate consistent improvement in child poverty since 1989 [29]. The multidimensional measures demonstrate rural children are more vulnerable to developmental risks than urban children [51]. For children affected by migration, left-behind children in rural areas show more developmental deficits than migrant children in urban areas, although the well-being of migrant children is behind their urban counterparts in various indicators [51].

Several limitations of child well-being measurements for China are noted. Cultural conceptualizations of well-being that differ across countries is difficult to capture especially in comparative studies that strive to use the same indicators across all countries. Nor do these studies capture the interactions among the dimensions that reinforce well-being. Even regional comparisons within countries may reflect the dominant culture and omit minority perspectives on well-being. Second, an issue in all of the measures is the lack of consistent child-centered indicators, particularly for economic well-being. For example, the Child Prosperity Index [50] depicts the general context of the country rather than the condition of children (13 out of 18 indicators used are non-child specific). Likewise, the UNICEF report uses child-centered indicators in the health, education, and safety dimensions, but the economic well-being and living environment dimensions are household- rather than child-centered measures. Finally, not all the studies use regularly collected data that does not allow for trends to be demonstrated.

The Chinese government has also shown interest in developing multidimensional measures to capture child well-being in China. Since 1992, The Government of China has adopted a periodic plan, the National Programme of Action for Children (NPA), to improve child development and promote children’s rights. NPA plans were implemented in 1992, 2001, and 2011. The plans target improvements in the various dimensions of child well-being, such as health, education, child welfare, living environment, and legal protection as goals. The NPA also established indicators to evaluate the progress in promoting child well-being and an ongoing system to monitor progress. The monitoring system is led by the National Bureau of Statistics (NBS) who the collects and analyzes data, some of which is published through the statistical yearbooks and annual statistical bulletins of relevant ministries under the State Council, such as China Statistical Yearbook, China Health and Family Planning Statistical Yearbook, and National Educational Development Statistical Bulletin. The NBS also established the Comprehensive Statistical Report System for Women and Children’s Conditions in 2004 at the national and provincial levels to improve information on gender equality and child well-being [52]. Highlights of the findings are publicly available.

Efforts have also been made to develop regional/provincial composite indices to monitor the trends of child well-being over time. For example, Zhejiang Province has developed a Key Developmental Indicator (KDI) Index to evaluate its progress in promoting child development [53]. This index has four components: health and protection, education and training, legal protection and environment, and a public satisfaction evaluation. The majority of the indicators are in accordance with the goals and key indicators of the NPA, but some indicators also reflect the concerns of the local government, such as food and drug safety and the quality of children’s products. The first KDI report was released in 2017. It evaluates the changes in children’s conditions in Zhejiang Province in 2014–2015. The regional-level indices and rankings are also available for comparisons across the 11 prefecture-level divisions. The report, however, lacks indicators on economic well-being and safety conditions for children. 

Next we considered the international multidimensional child well-being indices we reviewed earlier [17,49,54,55] and determined indicators to be included for the China child well-being index. Included are indicators that have data available in China and are culturally appropriate. We summarize the availability of potential data for indicators in each dimension and the sources below.

Thirty-four indicators were identified for six dimensions: economic well-being, physical health, emotional health and social relationships, education, safety and risk behaviors, and living environment. Table 2 lists the name, source, and frequency of data collection for each of the indicator. Data are drawn from administrative data (e.g., NPA Monitoring Statistics, China Statistical Yearbook, and Educational Statistics Yearbook of China) and nationwide sampled survey (e.g., CFPS and CNHSS). Most data sources collect data annually or biannually. Both positive and negative indicators are included in order to promote a holistic and comprehensive assessment of child well-being. For example, the physical health dimension not only contains negative indicators, such as mortality rate, low birth weight, and malnutrition, but also measures positive indicators that may be beneficial for children’s health (e.g., exercise frequently, exclusive breastfeeding, and health care administration). In addition, the economic well-being dimension takes into account the employment status of parents, a positive vision of family’s economic involvement and opportunities.

As demonstrated in Table 2, data availability varies across the indicators identified. Three indicators are regularly available to assess economic well-being. For the dimensions of education and physical health, there are a number of diverse, annually collected indicators, in contrast to indicators of mental health. Three of the four mental health indicators are not collected annually and are available only for children 10–15 years old. Evidence of prevalence in a population is often a good starting point to build services and unless measures of children’s emotional well-being are assessed, children may be deprived of services that would help them realize their full potential. Similarly, indicators of safety and risk are limited regarding the types of risks that children experience. The indicators do not consider substances other than alcohol that may be abused by children including cigarette smoking. This is an example of how interviews or focus groups with children could help reveal risk taking behaviors that are not known or publicly acknowledged such as HIV/AIDS. It would be helpful to know what makes children, at different ages, feel safe in their environments. Additional indicators should also be added to the Living Environment dimensions such as levels of pollutants and traffic congestion. It would be interesting to learn how children value privacy, family time and access to playgrounds for example.

## 5. Discussion

Given the data sources available and previous efforts, we believe that it is possible for China to develop its own multidimensional measure of child well-being that reflects positive and negative indicators of well-being. Our proposal is that future efforts should reflect and incorporate the interdependence and interaction of these dimensions on child well-being. Like the SDGs, multidimensional measures of child well-being interact with one another and are fundamentally interdependent. Understanding the range of positive and negative interactions among the different dimensions is key to unlocking their full potential and to ensuring that progress made in some areas is not made at the expense of progress in others. Policies and programs can determine the interactions and synergies of the dimensions. At a conceptual level, this makes sense yet, to date, accurately capturing the interdependence of these dimensions remains a challenge. How does one measure the effects of effective and accessible governance on children’s health or social well-being? Conceptually the linkages are there but more work needs to be done on establishing valid and reliable measures.

Efforts going forward should begin by advocating for solid data to be regularly collected in the dimensions we know affect child well-being. Moore [58] offers these guidelines for the collection of data on indicators: available over time; easy to interpret and disseminate; reflect social goals for children; cost effective; and well accepted for interpretation; and robust and statistically valid. Indeed the need for the indicators to be culturally and politically relevant should be underscored. Independent decision-making may be desirable and interpreted as mastering a developmental milestone in one culture and viewed as disrespectful in another.

China’s NPA makes explicit China’s goals for its children. The indicators collected should be tied to the NPA or other officially sanctioned goals for children in order to better understand areas of needed progress. Included should be indicators of children’s emotional well-being and children’s perception of their well-being. Except for the study by Chen and colleagues [51], indicators of emotional well-being were omitted from the other studies. A study conducted in northeastern China reveals that approximately one in 10 school children has DSM-IV disorders, and 15.2% of them have two or more comorbid disorders [59]. One in five children ages 10–15 reported having depression symptoms more than two times a week in 2010 [51]. It would be important to know more about the mental health effects of migration on children. The consequence of poor mental health status can be profound and greater attention should be made to this dimension.

Previous studies have shown that both migrant and left-behind children had poorer psychological and behavioral outcomes than local children [60,61]—calling for further depiction of indicators reflecting the emotional well-being of children, especially for children affected by migration.

Since China’s demographic structure differs from other countries largely because of the One Child policy that was in place for decades, the prevalence of child poverty may be higher in certain households because families with more than one child were often punished economically for multiple births, or a different child poverty pattern may exist. Poverty among urban children may be underestimated because of migration and ineligibility for social welfare benefits.

As this study points out, there are a number of methods to assess the multidimensional well-being of children. Each have strengths and weakness to consider. Researchers have developed measurements to assess child poverty through deprivation [62,63,64,65]. Alternatively, countries are increasingly adapting the Alkire-Foster methodology.

Our recommendation is that a combination of positive and negative indicators should be used when developing an index. The benefits of focusing on factors that promote children’s well-being rather than threatening their survival are numerous and focusing solely on how and when children do not fare well is unbalanced and contributes to public perceptions that taxpayer dollars are exclusively spent on children whose individual or family problems are intractable thus undermining public investments in all children [66]. Positive indicators can provide evidence needed to demonstrate the effectiveness of social policies implemented to improve the well-being of children. Emphasizing the strength (through positive indicators) rather than failures (through negative indicators) of communities is more likely to empower individuals and communities to work toward policy goals rather than disenfranchise them. Positive indicators are more likely to capture the goals and values of a society and promote prevention rather critique contemporary society [67].

Negative indicators are more likely to grab the attention of the public and the media and to motivate the calls for action that ensue. Negative indicators, such as increased crime, are more likely to facilitate additional public resources than reports demonstrating that investments in youth’s health have been successful [66].

However, recent studies have shown that positive indicators do resonate at the community level and among service providers. For example, the finding that when families eat dinner together their children have better outcomes can now be found on placards in subways, in grocery stores and radio public announcements. This type of social marketing based upon positive indicators can change family behaviors and produce more positive outcomes for children [68,69].

We also strongly believe that the construction of a multidimensional index should include the voices of children and families. Children and families should be interviewed either individually or in focus groups about what they believe to be indicators of child well-being. Data collected from these interviews would reflect cultural values unique to China and those that China shares with other countries. The child’s perspective may also help policymakers understand which policies resonate with children and families today.

## 6. Conclusions

We recommend that the multidimensional measure explicitly include children’s perspectives and state the understood interactions of the different dimensions regarding child well-being. Given budgetary, political, and resource constraints, as well as specific needs and policy agendas, countries are likely to prioritize certain goals, targets, and indicators over others. We need to better understand the interlinkages of these factors to help policymakers faced with limited resources decide which of the goals should realistically be tackled first and how. We suggest building on the framework, Nilsson, Griggs, and Visbeck (70) developed to assess the interlinkages of SDGs among one another [70]. In Nilsson et al.’s framework, progress on SDG goals are rated on the scale below and based on data presented. Table 3 summarizes this framework. For example, the dimension of economic well-being would be evaluated against the six other dimensions of child well-being. The dimensional interactions are critical and further knowledge is needed about these interdependencies and processes. This kind of information would be especially useful in China where market, family and government roles are being redefined. We offer the following as a preliminary exercise to begin to understand these interactions.

For example, the number of children affected by migration increased significantly over the past 20 years. In 2010, approximately 106 million children were affected by migration, comprising about 38% of the child population in China, which was almost 2.5 times of the number in 2000 [42,43,44]. However, we continue to lack information on how different dimensions affect the well-being of migrant children. About one-fourth of migrant children and 12% of left behind children are not attending senior secondary education [45]. It would be helpful to know how education intersects with income, housing, health, etc.

Indicators on child protection and safety should be included. Child safety has garnered increasing attention from the public recently. Even though the child injury-related mortality rate (ages under 18) was decreased from 22.4 in 2010 to 15.82 (per 100,000 children) in 2015 [71], children are still vulnerable to various types of risk factors, such as trafficking and violent crime. Between 2009 and 2013, it is estimated that there were 13,723 trafficking cases involving children [44]. The actual number is most likely higher since each case is likely to involve more than one child [44]. In addition to human trafficking, the safety of children is also violated by various types of abuse and maltreatment. A systematic review of 68 studies estimates that over one out of four (26.6%) children under 18 in China have suffered physical abuse, and 8.7% have suffered sexual abuse [72]. According to the calculation by Girl’s Protecting, the total number of child sexual abuse cases (under age 14) reported by the media were 433 in 2016, which equals to 1.21 cases a day [73]. The literature on the prevalence of child abuse and its effects on children’ emotional health and risk behaviors in China [74,75,76,77] has grown in recent years, but official indicators on child protection and how they relate to other dimensions are needed.

Though not complete, there is sufficient disaggregated data to develop a child well-being index in China that will both inform policy-making and monitor the effectiveness of policy decisions on all and different groups of children.

## Figures and Tables

**Table 1:** Dimensions and characteristics used in child well-being measurements for China.

Dimensions	Child Well-Being in the Pacific Rim—Lau and Bradshaw [49] ^1^	Child Prosperity Index across G20 Countries—Save the Children [50]	Children in China: An Atlas of Social Indicators—UNICEF [44]	Report on the State of Children in China—Chen, Yang, and Ren [51]	Multidimen-Sional Child Poverty Index—Qi and Wu [29]
Economic well-being	√	√	√	√	√
Home environment					
Community context	√	√	√	√	√
Family context	√	N/A	N/A	√	N/A
Safety/risk behaviors	√	√	√	N/A	N/A
Health	√	√	√	√	√
Education	N/A	√	√	√	√
Emotional/subjective well-being	√	N/A	N/A	√	N/A
Social relationships	N/A	N/A	N/A	√	N/A
Gender equality	N/A	√	N/A	N/A	N/A
Characteristics					
Children’s perspective	√	N/A	N/A	√	N/A
Construction of indicators	Composite index	Composite index	Individual indicators	Individual indicators	Composite index
Data type & source	Aggregated (international sampled surveys and databases)	Aggregated (international sampled surveys and databases)	Aggregated (national administrative data and regional sampled surveys)	Micro-data (China Family Panel Studies)	Micro-data (China Health and Nutrition Survey)
Age range	0–19	0–19 ^2^	0–17 ^2^	0–15	0–18
Child-centered indicators used	All dimensions, except income poverty and facilities component.	Life expectancy at birth, child mortality rate, overweight and obesity prevalence, PISA scores, youth unemployment.	Most indicators in the health, education, and safety/risk behaviors dimensions.	All dimensions, except some indicators in the economic and community context dimensions.	All indicators measure whether children are deprived in a certain condition.

^1^ Review for this study is based on dimensions, components, and indicators with data potentially available for China; ^2^ Data is generally for listed age range but some indicators’ age range are beyond the range, e.g., 15–24 years.

**Table 2:** China child well-being index: Indicators, potential sources, and characteristics.

Dimension/Indicators	Source	Frequency of Collection	Notes
Economic Well-Being			
Child poverty rate (under 18)	China Family Panel Studies (CFPS)	Bi-annually	CFPS sampled in 25 provinces, even though these provinces account for 95% of the Chinese population; reliability of self-report; secure parental employment rate is not a child-centered indicator.
Median annual income (all families with children under 18)			
Secure parental employment rate ^1^ (all families with children under 18)			
Physical Health			
Infant and under-5 mortality rate	China Health and Family Planning Statistical Yearbook (CHFPSY)	Annually	Data are drawn from the Maternal and Child Health Monitoring System covering 334 districts and counties around the nation [31].
Mortality rate (age 1–19)	China Population and Employment Statistics Yearbook	Annually	
Low birth weight	CHFPSY	Annually	
Immunization and vaccination rates (under 7)	NPA monitoring Statistics	Annually	Data available on each vaccine and immunization coverage.
Exclusive breastfeeding (<6 months)	NPA monitoring Statistics	Annually	
Moderate and severe malnutrition, under−5	CHFPSY	Annually	Lack of nutrition status for older children.
Underweight, under−5	NPA monitoring Statistics	Annually	
Obesity (age 7–17)	China Nutrition and Health Surveillance System (CNHSS)	Every 4–5 years	Relatively low frequency of data collection.
Exercising frequently (age 6–17) ^2^	CNHSS	Every 4–5 years	Relatively low frequency of data collection.
System administration rate of children under 3 years old	CHFPSY	Annually	
Health care administration rate of children under 7 years old	CHFPSY	Annually	
Rate of Children with health insurance (both public and private insurances) (age 0–15)	CFPS	Bi-annually	Reliability of parent-report.
Emotional Health and Social Relationships			
Suicide rate (age 0–15 or 0–20)	CHFPSY	Annually	Data are divided by urban and rural areas, gender specific data are available for both urban and rural areas.
Depression (%) (age 10–15)	CFPS	Bi-annually	Not a diagnostic result; only covers 10–15 age group.
Lack good personal relations (age 10–15)	CFPS	Bi-annually	Reliability of self-report; only covers 10–15 age group.
Lack good social skills (age 10–15)	CFPS	Bi-annually	Reliability of self-report; only covers 10–15 age group.
Education			
Gross enrolment ratio in three-year pre-primary education (age 3–5)	Educational Statistics Yearbook of China	Annually	
Cohort survival rate in 9-year compulsory education ^3^	National Educational Development Statistical Bulletin (NEDSB)	Annually	
Promotion rate of junior secondary school graduates	Educational Statistics Yearbook of China	Annually	
Number of children affected by migration enrolled in 9-year compulsory education ^4^	China Statistical Yearbook	Annually	
Mean test scores of vocabulary and math (age 10–15)	CFPS	Bi-annually	Measurement is developed by the study, not a national standardized test.
Mean test scores of math (age 10–15)	CFPS	Bi-annually	Measurement is developed by the study, not a national standardized test.
Safety and Risk Behaviors			
Adolescent fertility rate (per 1000 women) (age 15–19)	China Statistical Yearbook	Annually	
Injury-related death rate (under 18)	NPA Monitoring Statistics	Annually	
Rate of alcohol drinking (age 15–17)	CNHSS	Every 4–5 years	Relatively low frequency of data collection.
Proportion of juvenile delinquents among criminal offenders (under 18)	NPA Monitoring Statistics	Annually	
Living Environment			
Percentage of population benefiting from drinking water improvement in rural areas	CHFPSY	Annually	Not child-centered indicator; lack of information for urban areas.
Access rate to sanitary toilets in rural areas	CHFPSY	Annually	Not child-centered indicator; lack of information for urban areas.
Parents communicate with child (age 6–15)	CFPS	Bi-annually	Reliability of parent-report.
Parents read to child (age 3–5)	CFPS	Bi-annually	Reliability of parent-report.
House crowding for 0–15 year olds or 0–18 year olds	CFPS	Bi-annually	Not child-centered indicator; lack of children’s subjective feelings.

^1^ Secure parental employment rate is defined as “at least one parent employed full time all year” [56] (p. 7); ^2^ Exercising frequently is defined as exercising at least three times a week and more than 30 minutes each time; ^3^ Cohort survival rate in nine-year compulsory education is the percentage of children enrolled in the first grade of primary school who eventually reach the third grade of junior secondary school [57]. ^4^ Children affected by migration include children migrant with parents and children left behind.

**Table 3:** Rating interlinkage effects among dimensions ^1^.

Indivisible	Reinforcing	Enabling	Consistent	Constraining	Counteracting	Cancelling
+3	+2	+1	0	−1	−2	−3
One dimension is highly interlinked with another. Strong positive relationship.	One dimension directly creates conditions that lead to the implementation of another objective.	Pursuing one dimension facilitates the betterment of another dimension.	Neutral relationship among dimensions. One dimension is unlikely to affect the other.	Mildly negative relationship. Improvement in one dimension may negatively affect the other dimension.	Improving one objective negatively affects the other dimension.	Furthering one dimension clearly diminishes progress in the other dimension.

^1^ Adapted from Nilsson, M.; Griggs, D.; Visbeck (70).
